# Adherence to the Recommended HPV Vaccine Dosing Schedule among Adolescents Aged 13 to 17 Years: Findings from the National Immunization Survey-Teen, 2019–2020

**DOI:** 10.3390/vaccines10040577

**Published:** 2022-04-08

**Authors:** Chinenye Lynette Ejezie, Ikponmwosa Osaghae, Sylvia Ayieko, Paula Cuccaro

**Affiliations:** 1 Department of Health Promotion and Behavioral Sciences, The University of Texas School of Public Health, 1200 Pressler St, Houston, TX 77030, USA; sylvia.a.ayieko@uth.tmc.edu (S.A.); paula.m.cuccaro@uth.tmc.edu (P.C.); 2 Department of Investigational Cancer Therapeutics, The University of MD Anderson Cancer Center, Houston, TX 77030, USA; 3 Department of Biostatistics, The University of Texas MD Anderson Cancer Center, Houston, TX 77030, USA; ikponmwosa.osaghae@uth.tmc.edu; 4 Department of Epidemiology, Human Genetics & Environmental Sciences, The University of Texas School of Public Health, Houston, TX 77030, USA

**Keywords:** HPV vaccination, dosing schedule, dosing interval, 9-valent, low adherence

## Abstract

The 9-valent human papillomavirus (9-vHPV) vaccine uptake rate among adolescents has improved over the years; however, little is known about the adherence to the recommended dosing schedule. This study examines the prevalence and factors associated with adherence to the recommended 9vHPV vaccination dosing schedule among adolescents aged 13 to 17 years. The cross-sectional study was conducted using the 2019–2020 National Immunization Survey-Teen. The parents of 34,619 adolescents were included in our analyses. The overall up-to-date (UTD) prevalence was 57.1%. The UTD prevalence was 60.0% among females and 54.2% among males. Adolescents aged 16 years had the highest UTD prevalence of 63.0%. The UTD prevalence was 61.6% among Hispanics and 54.7% among non-Hispanic Whites. Overall, compared to females, males had 14% lower odds of UTD. The odds of UTD were 1.91 times, 2.08 times, and 1.98 times higher among adolescents aged 15–17 years, respectively, compared to those aged 13 years. Moreover, region, poverty, insurance status, mothers’ educational level, and provider recommendation were associated with UTD. Our findings show that adherence to the recommended 9vHPV vaccine schedule is low in the US. Targeted public health efforts are needed to improve the rates of adherence to the recommended 9vHPV dose schedule.

## 1. Introduction

In the United States, the Advisory Committee on Immunization Practices (ACIP) and the Centers for Disease Control and Prevention (CDC) recommend that adolescents prophylactically receive the 9-valent human papillomavirus (HPV) vaccine (9vHPV) [1,2,3,4]. Toward the end of 2016, the 9vHPV vaccine became the only HPV vaccine available in the US and the administration of other types of HPV vaccines were permanently discontinued [5]. Clinical trials have shown that 9vHPV is about 90% effective in preventing HPV-related cancers when administered on the recommended dosing schedule [6,7,8,9]. The dose schedule of 9vHPV differs across age categories: the ACIP and CDC recommends two doses administered at a schedule of 5 to 12 months for persons younger than 15 years [10,11]. The CDC recommends a third dose for people younger than 15 years who receive the second dose less than 5 months following the first dose [10,11]. Additionally, the CDC recommends three doses (second dose administered at a schedule of 1 to 2 months after the first dose; and the third dose administered 6 months after the first dose) for persons who initiate the vaccine series on or after their 15th birthday, as well as those with a compromised immune system [11]. 

Despite the recommendation by the ACIP and CDC, the number of adolescents adhering to the recommended dosing schedule remains relatively low. Adherence to the recommended dosing schedule is crucial to optimize immune response to the HPV vaccine [12,13]. According to the CDC, only about half of adolescents aged 13 to 17 years adhere to the recommended HPV vaccine dosing schedule [1]. A possible contributing factor of low adherence to the recommended dosing schedule is little or no emphasis on the dosing schedule in reporting the vaccine series as complete [14,15,16]. Specifically, the vaccine is often categorized as complete based on the uptake of the required number of doses (two or three doses depending on age category) without considering the interval between doses [14,15,16]. For example, in Munn et al.’s study conducted to examine the uptake and completion of HPV vaccine series among adolescent users and nonusers of school-based health centers, completion was defined as the uptake of the required number of doses per age category, with no information about the interval between doses [15]. Similarly, another study conducted by Simons et al. to estimate and examine predictors of HPV vaccine series completion defined completion based on the receipt of three doses of HPV vaccine within one year without considering adherence to the recommended dosing schedule [14]. Given that adherence to the recommended number of doses and interval between doses is necessary for efficacy [17,18], considerations should be made to note both requirements when categorizing HPV vaccination as complete.

Observational studies have identified several factors that influence the uptake of the HPV vaccine among adolescents [13,14,15]; however, the factors that influence adherence to the recommended dosing schedule remain unexplored. It is plausible that the factors associated with HPV vaccine uptake are also associated with adherence to the recommended HPV dosing schedule. For example, sociodemographic characteristics, including age, race/ethnicity, income level, insurance status, census region, and sex; and factors such as provider recommendation, parental hesitancy, structural barriers, and knowledge influence HPV vaccine uptake [13,14,15,16,19] and, therefore, may affect adherence to the recommended dosing schedule.

Receiving two or three doses of 9vHPV is not enough to offer the expected protection from HPV-related diseases [10,18]. Complying with the recommended interval between doses is also essential to ensure HPV vaccine efficacy [10,17,18]. Since adherence to the recommended dosing schedule seems necessary for protection from HPV-related infections [10,17,18], the efficacy of the 9vHPV vaccine may be reduced when the intervals between doses are not according to the recommended schedule. Adverse effects associated with not receiving the HPV vaccine, such as risk of HPV-related cancers [10], may be associated with non-adherence to the recommended HPV vaccine dosing schedule. Although previous research has shown that HPV vaccine uptake among adolescents aged 13 to 17 years has improved over the years, limited research on HPV vaccine completion has examined the adherence to the recommended dosing schedule. Thus, the goal of this research is to examine the prevalence and factors associated with adherence to the recommended HPV vaccination dosing schedule among adolescents aged 13 to 17 years. Findings could help researchers and policy makers to improve adherence to the recommended dosing schedule by highlighting the importance of dosing intervals when creating programs aimed at improving HPV vaccination.

## 2. Materials and Methods

Study Design, Setting, and Participants

In this secondary analysis, we utilized the 2019–2020 National Immunization Survey Teen (NIS-Teen) conducted by the CDC [20]. The NIS-Teen is conducted annually with samples of parents or caregivers of adolescents who are aged 13 to 17 years and reside in the US. The sampling frame includes a representative sample of eligible parents with landlines or cell phones. The survey consists of provider-verified data on vaccines from adolescents aged 13 to 17 years in the 50 states, the District of Columbia, and territories.

The survey occurs in two phases: in phase 1, parents or caregivers are contacted via telephone to provide information pertaining to their adolescents’ sociodemographic characteristics, contact information, and vaccination history. Additionally, parents or caregivers are asked to provide consent for the adolescents’ healthcare provider to be contacted. In phase 2, healthcare providers are contacted through a mailed survey to verify the accuracy of the information obtained from parents or caregivers. Detailed information about the methods for the NIS-Teen study are available elsewhere [20].

## 3. Measures

### 3.1. Dependent Variable

#### HPV Vaccine Uptake

The dependent variable assessed whether an adolescent adhered to the required dosing schedule when completing the HPV vaccine series. Specifically, the variable assessed whether adolescents were HPV vaccine up-to-date (UTD) in line with the required dosing and interval between HPV vaccine shots. This variable was first included in the NIS-teen survey in 2016. UTD was defined as 3+ human papillomavirus shots (9V, 4 V, UV, CV, or HP) or 2+ human papillomavirus shots, with the first shot received before age 15 and an interval between first and second shots of at least 5 months (minus 4 days), excluding any vaccinations after the random digit dialing interview date [20]. This variable was binary, i.e., “UTD” versus “Not UTD”.

### 3.2. Independent Variables

#### Provider Recommendation

To assess provider recommendation, respondents were asked, “Had or has a doctor or other health care professional ever recommended that teen receive HPV shots?” Responses were either “Yes”, “No”, “Don’t Know”, or “Refused”. This variable was operationalized as binary, with only responses “Yes” or “No” retained for our analysis.

### 3.3. Sociodemographic Characteristics

Sociodemographic characteristics assessed based on previous literature were adolescent’s age (categorical variable 13–17 years), gender (male or female), race/ethnicity (non-Hispanic White, non-Hispanic Black, Hispanic, and non-Hispanic Other), region (West, Midwest, Northeast, and South), and insurance status (any Medicaid, private insurance only, other insurance, and uninsured). Other sociodemographic characteristics assessed were poverty status, defined as percentage of poverty line (categorized as above poverty ≤USD 75 k, above poverty >USD 75 k, and below poverty), and mother’s education status (categorized as college graduate, some college, high school only, and less than high school).

### 3.4. Statistical Analysis

All analyses were conducted using Stata/IC V.15.1. The analyses accounted for the complex study design and survey sampling weights used in the NIS-Teens survey. The inclusion criterion was the presence of adequate provider data for UTD and not UTD adolescents. We excluded all “Don’t Know” and “Refused” responses for provider recommendation, and “Unknown” responses for poverty status. A complete case analysis was conducted; as such, respondents with missing data for provider recommendation (1.79%) and region (0.84%) were excluded from the analysis. Weighted percentages were reported and are, therefore, representative of the general population. Descriptive statistics were presented in the overall population and in populations stratified by gender using simple proportions and chi-square test. Furthermore, we presented the weighted prevalence of UTD by sociodemographic characteristics and provider recommendations in the overall population and stratified by gender. Bivariable and multivariable logistic regression analyses were used to estimate the association between sociodemographic characteristics and provider recommendation with HPV UTD among adolescents aged 13–17 years in our overall study sample and separately among females and males. Each model was adjusted for sociodemographic characteristics, provider recommendation, and survey year. Statistical significance was defined as a two-sided *p*-value < 0.05 for all comparisons.

## 4. Results

### 4.1. Overall Population

A total of 34,619 adolescents were included in our final analyses. Among those who were UTD in the overall population, most were females (51.8%), 16 years old (22.0%), non-Hispanic White (50.7%), resided in the southern region (36.5%), had private insurance only (55.0%), had income above poverty >USD 75 k (51.0%), had mothers who were college graduates (45.8%), and had received a provider recommendation (88.9%) (Table 1).

The overall UTD prevalence among all adolescents was 57.1% (95% CI: 56.0–58.1%). In the overall population, the UTD prevalence was 60.0% among females and 54.2% among males. Adolescents aged 16 years had the highest UTD prevalence of 63.0%. The UTD prevalence was also highest (61.6%) among Hispanics and highest (63.9%) among adolescents residing in the Northeast region. Moreover, adolescents who had any Medicaid had the highest (60.7%) UTD prevalence, while adolescents below the poverty line had the highest (60.8%) UTD prevalence. The UTD prevalence was highest (61.7%) among adolescents with mothers having less than high school education and highest (62.8%) among adolescents who received a provider recommendation. Additionally, the UTD prevalence was 54.6% in 2019 and 59.5% in 2020 (Table 2).

In the overall population, results of multivariable regression analysis showed that, compared to uninsured adolescents, adolescents who had any Medicaid had over twofold higher adjusted odds of UTD (AOR: 2.13; 95% CI: 1.64–2.76). Moreover, compared to adolescents below the poverty line, those living above poverty <= USD 75 k had 22% lower adjusted odds of UTD (AOR: 0.78; 95% CI: 0.66–0.92). Adolescents residing in the Midwest and Northeast regions had 1.19- and 1.49-times higher odds of UTD, respectively, compared to those residing in the Southern region (AOR: 1.19; 95% CI: 1.08–1.31, AOR: 1.49; 95% CI:1.33–1.66, respectively). Additionally, adolescents who received a provider recommendation had about twofold higher adjusted odds of UTD compared to those who did not (AOR: 1.85; 95% CI: 1.74–1.97). We also found 22% higher odds of UTD in 2020 compared to 2019 (Table 2).

### 4.2. Female Adolescents

Following stratification by gender, among females, the UTD prevalence was 60.0% (95% CI: 58.5–61.5%). Moreover, UTD prevalence was 63.2% among female adolescents on any Medicaid and 44.8% among uninsured females. Female adolescents below the poverty line had a UTD prevalence of 62.9%, while those above the poverty line ≤USD 75 k had a UTD prevalence of 56.4%. Female adolescents who received a recommendation from a provider had a UTD prevalence of 64.8%, while those who received no provider recommendation had a UTD prevalence of 34.2% (Table 3).

Among females, results of multivariable regression analysis showed that, compared to female adolescents that are uninsured, those with any Medicaid, private insurance only, and other insurance had 124%, 68%, and 62% higher adjusted odds of UTD, respectively. Moreover, females who had received a provider recommendation had 92% (AOR: 1.92; 95% CI: 1.75–2.11) higher adjusted odds of UTD compared to those who received no recommendation (Table 3).

### 4.3. Male Adolescents

Following stratification by gender, among males, the UTD prevalence was 54.2% (95% CI: 52.7–55.7%). Male adolescents who had any Medicaid insurance had the highest (58.3%) UTD prevalence, while those who were uninsured had the lowest (39.6%) UTD prevalence. Male adolescents below the poverty line had a UTD prevalence of 58.8%, while those above the poverty line >USD 75 k had a UTD prevalence of 54.0%. Male adolescents who received a recommendation from a provider had a UTD prevalence of 60.8%, while those who received no provider recommendation had a UTD prevalence of 31.9% (Table 3).

Furthermore, among male adolescents, results of multivariable logistic regression analysis were mostly similar but slightly attenuated compared to what was seen among female adolescents. Male adolescents with any Medicaid had 102% higher adjusted odds of being UTD compared to those who were uninsured. In terms of poverty status, compared to male adolescents below the poverty line, those living above poverty ≤USD 75 k had 27% lower adjusted odds of UTD (AOR: 0.73; 95% CI: 0.58–0.92) (Table 3).

## 5. Discussion

To our knowledge, our study is among the first to employ the 2019–2020 NIS-Teen dataset to examine the prevalence and factors associated with the adherence to the ACIP recommended 9vHPV vaccine schedule among teenagers aged 13 to 17 years. Nationally, only about half of females and males completed the vaccine series with adherence to the recommended schedule. Our finding is consistent with previous research, which shows that only 58.6% of teenagers adhered to the recommended dosing schedule [1]. Without adherence to the recommended dosing schedule, immune response imparting expected protection from the HPV vaccine is uncertain [18]. It is possible that expected immune protection might be reduced or absent outside of the recommended vaccination schedule [9].

Our low adherence finding could be related to a number of possible reasons, including providers not scheduling follow-up doses at the time of the initial dose, or few or no reminders for follow-up dose scheduling. Since lack of knowledge is a factor associated with low HPV vaccine uptake [21,22,23], it could also be a reason for low adherence to the recommended vaccination schedule. Based on previous studies, knowledge is an important predictor of HPV vaccine uptake, and improvement in knowledge results in improvements in HPV vaccine behaviors [21,22,23]. Providing parents with information about the importance of adhering to the appropriate dosing interval may encourage parents to pay attention to the time points when vaccinating their adolescents. Moreover, creating intervention programs aimed at increasing knowledge on adherence to the recommended dosing may improve adherence to the recommended vaccination schedule.

In our provider recommendation analysis, we found that provider recommendation was consistently associated with HPV vaccine completion with adherence to the recommended dosing schedule. Our results are in agreement with other studies that have shown that parents are more likely to vaccinate their adolescents when they receive a recommendation from a healthcare provider [24]. How providers introduce and recommend vaccines is robustly associated with vaccine behaviors [25,26]. Specifically, “strong” provider recommendation (which encompasses the recommendation of same-day vaccination, emphasizing the completion of the vaccine series, and specifying the recommended dosing schedule) is associated with nine times the odds of HPV vaccine uptake compared to a weak recommendation [26]. Moreover, providing parents with information regarding the differences in dosing schedules and requirements by age provides an incentive for on-time vaccination [27]. Our provider-recommendation-related finding is possibly because parents who are rule followers adhere to the recommended schedule, or because parents who go to their primary care provider for vaccination are more likely to have a follow-up appointment scheduled and get a reminder call/postcard/text about their upcoming appointment. Although parental hesitancy discourages providers from having HPV-vaccine-related conversations with parents [25], it is important that providers understand that even hesitant parents are willing to change with provider encouragement. These findings offer early evidence for the need for provider education to improve the quality of provider recommendations, which could increase adherence to the recommended dosing schedule.

In our income analysis, we found that the odds of complying with the dosing schedule were higher among those below the poverty line. We found that adolescents who had any Medicaid had higher odds of adhering to the recommended HPV vaccine dosing schedule. According to Hoff et al. [28], Medicaid expansion resulted in a significant increase in HPV vaccine uptake among people living below the poverty level. Under the Vaccines for Children program (VFC), adolescents enrolled in Medicaid are eligible to receive all vaccines recommended by the ACIP at no cost to them or their families [29,30,31]. These factors may contribute to greater compliance with the vaccination schedule. Because VFC also covers uninsured adolescents, it is surprising that adherence to the recommended dosing schedule is lower in uninsured adolescents than in adolescents with only Medicaid. Therefore, the lower rates of adherence to the dosing schedule among uninsured adolescents should be investigated further.

In our census region analysis, we found that, in the South (where HPV vaccine completion is the lowest in the nation: Mississippi 28.8% and Wyoming 30.9%) [32], parents had lower odds of adhering to the recommended dosing schedule compared to other regions. This finding has important public health implications. If lower adherence to the recommended dosing schedule in the South census region persists, this region could face a disproportionately higher burden of HPV-related infections in future decades compared to regions with higher adherence to the recommended dosing schedule. Further research is needed to explore possible factors impacting adherence to the recommended HPV vaccine dosing schedule in the South census region and to determine how best to design interventions aimed at improving the adherence to the recommended HPV vaccine dosing schedule in the South census region.

While we found that the odds of vaccine completion with adherence to the recommended dosing schedule was higher in 2020 than in 2019, rates remain relatively low. Structural barriers, such as transportation difficulties, could make complying with the recommended vaccination schedule difficult for adolescents and their parents [33]. Thus, there is a need for improving vaccine accessibility for adolescents with parental consent by making the vaccine available in alternative settings, such as schools without the need for parents to be present. Increasing access to vaccination through systems-level interventions, such as school-based vaccination, can improve vaccination uptake with adherence to the vaccine schedule.

A limitation of this study is that the respondent is one parent/guardian of the adolescent and may not be the most knowledgeable about the adolescent’s health status and vaccinations. However, we addressed this limitation by including only respondents with adequate provider data. Another limitation is that our dependent variable was designed to account for adolescents who received the 9vHPV vaccine as well as those who may have received other types of HPV vaccines (4 V, UV, CV, or HP) prior to the discontinuation in 2017. While this limitation did not affect the goal of our study, which is to examine the adherence to the recommended dose schedule, there is a need for the NIS-Teen researchers to include a variable that focuses on the adherence to the dosing schedule of the 9vHPV vaccine. This strategy will allow HPV-vaccine researchers to better examine adherence to the recommended dosing schedule of the only HPV vaccine currently administered in the US. Our study may be prone to residual confounding from father’s educational status, as this variable is unavailable in the NIS-Teens dataset and was not accounted for in all our analyses. Other limitations, such as social desirability bias and recall bias, were also addressed by using respondents with adequate provider data. Strengths of the study include its large sample size and nationally representative data, and use of provider verified data.

Our study contributes to previous research by examining the sociodemographic factors associated with adherence to the HPV vaccine dosing schedule. Our findings suggest that adherence to the recommended dosing schedule remains relatively low. Adherence to the recommended dosing schedule is important for adequate immune response and expected protection from HPV infection. Therefore, findings from this research are important for the improvement in adherence to the HPV vaccine schedule.

## 6. Conclusions

It is important to investigate nonadherence to the recommended dosing schedule for HPV vaccination. Our cross-sectional analysis depicts salient factors, including provider recommendation, that can be targeted through interventions to improve adherence to the 9vHPV dosing schedule to offer better protection from HPV-related cancers. More research is needed to examine correlates of adherence to the recommended schedule for 9vHPV. Multilevel interventions to increase knowledge of the 9vHPV dosing schedule, improve the quality of provider recommendation, and remove access barriers to vaccination are warranted to improve the overall rates of adherence to the recommended 9vHPV dose schedule across races, age categories, and census regions.

## Figures and Tables

**Table 1:** Descriptive statistics of teens aged 13–17 years, stratified by gender, National Immunization Survey—Teen (2019–2020).

Characteristics	Overall Sample (*n* = 34,619)			Female (*n* = 16,623)			Male (*n* = 17,996)		
	Not UTD (*n* = 14,322) (*n* (w%))	UTD (*n* = 20,297) (*n* (w%))	*p*-Value	Not UTD (*n* = 6554) (*n* (w%))	UTD (n = 10,069) (*n* (w%))	*p*-Value	Not UTD (*n* = 7768) (*n* (w%))	UTD (*n* = 10,228) (*n* (w%))	*p*-Value
**Sex**			<0.001						
Female	6554 (45.8)	10069 (51.8)							
Male	7768 (54.2)	10228 (48.2)							
**Age, years**			<0.001			<0.001			<0.001
13	3799 (24.7)	3450 (15.3)		1826 (26.3)	1693 (16.3)		1973 (23.3)	1757 (14.2)	
14	3111 (21.8)	4168 (20.1)		1427 (22.4)	2031 (19.2)		1684 (21.3)	2137 (21.1)	
15	2631 (18.7)	4257 (21.7)		1186 (18.1)	2094 (22.3)		1445 (19.2)	2163 (21.1)	
16	2477 (17.2)	4432 (22.0)		1093 (16.3)	2238 (21.6)		1384 (17.9)	2194 (22.4)	
17	2304 (17.7)	3990 (20.9)		1022 (16.9)	2013 (20.7)		1282 (18.3)	1977 (21.2)	
**Race/ethnicity**			<0.001			<0.001			0.054
Non-Hispanic White	9589 (56.5)	12750 (50.7)		4395 (57.1)	6257 (50.1)		5194 (56.0)	6493 (51.4)	
Non-Hispanic Black	1035 (13.4)	1608 (13.7)		471 (13.8)	804 (13.2)		564 (13.1)	804 (14.2)	
Hispanic	2154 (20.8)	3579 (25.2)		966 (19.4)	1767 (25.4)		1188 (22.1)	1812 (25.0)	
Non-Hispanic Other	1544 (9.2)	2360 (10.4)		722 (9.7)	1241 (11.3)		822 (8.8)	1119 (9.5)	
**Region**			<0.001			0.002			<0.001
West	3365 (23.4)	4295 (24.5)		1546 (21.8)	2157 (25.2)		1819 (24.7)	2138 (23.7)	
Midwest	2986 (21.2)	4583 (21.4)		1315 (21.8)	2206 (21.0)		1671 (20.6)	2377 (22.0)	
Northeast	2255 (13.2)	4458 (17.6)		1068 (14.0)	2167 (16.7)		1187 (12.6)	2291 (18.5)	
South	5716 (42.3)	6961 (36.5)		2625 (42.5)	3539 (37.1)		3091 (42.1)	3422 (35.9)	
**Insurance status**			<0.001			<0.001			<0.001
Any Medicaid	3800 (30.9)	5803 (35.9)		1713 (30.3)	2857 (34.7)		2087 (31.4)	2946 (37.2)	
Private insurance only	8625 (56.3)	12558 (55.0)		3991 (57.5)	6240 (56.4)		4634 (55.3)	6318 (53.5)	
Other insurance	1252 (7.6)	1470 (6.3)		552 (7.1)	740 (6.2)		700 (8.0)	730 (6.4)	
Uninsured	645 (5.2)	466 (2.9)		298 (5.2)	232 (2.8)		347 (5.3)	234 (2.9)	
**Poverty status, % of poverty line**			<0.001			0.009			0.016
Above poverty ≤USD 75 k	4454 (33.3)	5444 (29.5)		2041 (33.6)	2639 (29.0)		2413 (33.1)	2805 (30.1)	
Above poverty >USD 75 k	7999 (50.1)	11867 (51.0)		3671 (49.5)	5910 (51.9)		4328 (50.6)	5957 (50.2)	
Below poverty	1869 (16.6)	2986 (19.4)		842 (16.9)	1520 (19.1)		1027 (16.4)	1466 (19.8)	
**Mother’s education status**			<0.001			0.001			0.058
College graduate	7004 (42.3)	10974 (45.8)		3205 (42.5)	5404 (46.7)		3799 (42.2)	5570 (44.8)	
Some college	4206 (26.4)	4982 (23.9)		1945 (26.9)	2464 (22.9)		2261 (26.0)	2518 (25.0)	
High school only	2140 (22.4)	2744 (19.6)		965 (22.2)	1390 (19.6)		1175 (22.6)	1354 (19.6)	
Less than high school	972 (8.9)	1597 (10.8)		439 (8.4)	811 (10.8)		533 (9.3)	786 (10.7)	
**Provider recommendation**			<0.001			<0.001			<0.001
No	4200 (30.2)	1932 (11.1)		1588 (25.7)	789 (8.9)		2612 (33.9)	1143 (13.5)	
Yes	10122 (69.8)	18365 (88.9)		4966 (74.3)	9280 (91.1)		5156 (66.1)	9085 (86.5)	
**Survey year**			<0.001			<0.001			0.009
2019	7294 (53.2)	9382 (48.2)		3325 (53.9)	4667 (47.9)		3969 (52.5)	4715 (48.4)	
2020	7028 (46.8)	10915 (51.9)		3229 (46.1)	5402 (52.1)		3799 (47.5)	5513 (51.6)	

n = unweighted number of participants; w%  =  weighted percentages. UTD = up-to-date was defined as 3+ human papillomavirus shots (9 V, 4 V, UV, CV, or HP) or 2+ human papillomavirus shots, with first shot received before age 15 and interval between first and second shots of at least 5 months (minus 4 days), excluding any vaccinations after the random digit dialing interview date.

**Table 2:** Prevalence and association between sociodemographic characteristics and provider recommendation among teens aged 13–17 years and UTD in the overall sample, National Immunization Survey—Teen (2019–2020).

Characteristics	Overall Sample (*n* = 34,619)		
	w% (95% CI)	Crude OR (95% CI)	Adjusted OR (95% CI) ^a^
**Overall**	57.1% (56.0–58.1%)		
**Sex**			
Female	60.0 (58.5–61.5)	Ref	Ref
Male	54.2 (52.7–55.7)	0.79 (0.72–0.86)	0.83 (0.76–0.91)
**Age, years**			
13	45.1 (42.9–47.3)	Ref	Ref
14	55.1 (52.6–57.6)	1.49 (1.30–1.71)	1.52 (1.32–1.75)
15	60.7 (58.4–62.9)	1.88 (1.64–2.14)	1.91 (1.67–2.19)
16	63.0 (60.7–65.2)	2.09 (1.84–2.37)	2.09 (1.82–2.40)
17	61.2 (58.5–63.7)	1.99 (1.73–2.27)	1.93 (1.66–2.23)
**Race/ethnicity**			
Non-Hispanic White	54.4 (53.2–55.6)	Ref	Ref
Non-Hispanic Black	57.5 (54.2–60.7)	1.13 (1.18–1.54)	1.22 (1.04–1.43)
Hispanic	61.6 (58.7–64.5)	1.35 (0.99–1.31)	1.51 (1.31–1.74)
Non-Hispanic Other	60.0 (57.0–63.0)	1.26 (1.10–1.44)	1.27 (1.11–1.46)
**Region**			
South	53.4 (51.9–55.0)	Ref	Ref
West	58.2 (55.1–61.3)	1.22 (1.05–1.40)	1.12 (0.97–1.31)
Midwest	57.4 (55.7–59.0)	1.17 (1.07–1.29)	1.19 (1.08–1.31)
Northeast	63.9 (61.8–65.8)	1.54 (1.39–1.71)	1.49 (1.33–1.66)
**Insurance status**			
Uninsured	42.1 (36.4–47.9)	Ref	Ref
Any Medicaid	60.7 (58.6–62.7)	2.13 (1.65–2.73)	2.13 (1.64–2.76)
Private insurance only	56.5 (55.1–57.8)	1.79 (1.40–2.28)	1.61 (1.23–2.09)
Other insurance	52.3 (48.4–56.2)	1.51 (1.14–2.01)	1.49 (1.10–2.01)
**Poverty status, % of poverty line**			
Below poverty	60.8 (58.0–63.6)	Ref	Ref
Above poverty ≤USD 75 k	54.1 (52.1–56.1)	0.76 (0.66–0.88)	0.78 (0.66–0.92)
Above poverty >USD 75 k	57.5 (56.1–58.9)	0.87 (0.77–1.00)	0.89 (0.73–1.08)
**Mother’s education status**			
College graduate	59.0 (57.6–60.4)	Ref	Ref
Some college	54.6 (52.5–56.7)	0.84 (0.75–0.93)	0.80 (0.71–0.89)
High school only	53.7 (50.9–56.4)	0.81 (0.71–0.91)	0.76 (0.65–0.88)
Less than high school	61.7 (57.7–65.4)	1.12 (0.94–1.33)	1.02 (0.82–1.28)
**Provider recommendation**			
No	32.8 (30.5–35.3)	Ref	Ref
Yes	62.8 (61.7–64.0)	1.86 (1.75–1.97)	1.85 (1.74–1.97)
**Survey year**			
2019	54.6 (53.0–56.2)	Ref	Ref
2020	59.5 (58.2–60.9)	1.22 (1.12–1.34)	1.22 (1.11–1.33)

w% = weighted percentages; OR = odds ratio; CI = confidence interval; Ref = reference category. UTD = up-to-date was defined as 3+ human papillomavirus shots (9 V, 4 V, UV, CV, or HP) or 2+ human papillomavirus shots, with first shot received before age 15 and interval between first and second shots of at least 5 months (minus 4 days), excluding any vaccinations after the random digit dialing interview date. ^a^ Model adjusted for sociodemographic characteristics, provider recommendation, and survey year.

**Table 3:** Prevalence and association between sociodemographic characteristics and provider recommendation among teens aged 13–17 years and UTD, stratified by gender, National Immunization Survey—Teen (2019–2020).

Characteristics	Female (*n* = 16,623)				Male (*n* = 17,996)	
	w% (95% CI)	Crude OR (95% CI)	Adjusted OR (95% CI) ^a^	w% (95% CI)	Crude OR (95% CI)	Adjusted OR (95% CI) ^a^
**Overall**	60.0 (58.5–61.5)			54.2 (52.7–55.7)		
**Age, years**						
13	48.1 (44.8–51.4)	Ref	Ref	41.8 (38.9–44.7)	Ref	Ref
14	56.3 (52.7–59.8)	1.39 (1.14–1.69)	1.40 (1.14–1.71)	53.9 (50.4–57.5)	1.63 (1.35–1.97)	1.65 (1.36–1.99)
15	64.8 (61.8–67.8)	1.99 (1.65–2.40)	2.06 (1.70–2.50)	56.5 (53.2–59.8)	1.81 (1.51–2.17)	1.80 (1.49–2.18)
16	66.5 (63.2–69.7)	2.14 (1.76–2.60)	2.14 (1.74–2.64)	59.7 (56.6–62.8)	2.06 (1.73–2.46)	2.05 (1.72–2.45)
17	64.8 (61.1–68.4)	1.98 (1.61–2.44)	2.00 (1.64–2.44)	57.7 (54.0–61.4)	1.90 (1.57–2.30)	1.87 (1.52–2.30)
**Race/ethnicity**						
Non-Hispanic White	56.8 (55.1–58.6)	Ref	Ref	52.0 (50.4–53.7)	Ref	Ref
Non-Hispanic Black	59.1 (54.4–63.6)	1.10 (0.89–1.34)	1.21 (0.97–1.52)	56.0 (51.4–60.5)	1.17 (0.96–1.43)	1.22 (0.98–1.51)
Hispanic	66.3 (62.3–70.0)	1.49 (1.24–1.80)	1.67 (1.36–2.05)	57.2 (53.0–61.4)	1.23 (1.03–1.48)	1.39 (1.13–1.70)
Non-Hispanic Other	63.6 (59.5–67.6)	1.33 (1.10–1.60)	1.33 (1.10–1.60)	56.0 (51.7–60.3)	1.17 (0.97–1.42)	1.22 (1.00–1.48)
**Region**						
South	56.7 (54.5–58.8)	Ref	Ref	50.2 (48.0–52.4)	Ref	Ref
West	63.5 (59.0–67.8)	1.33 (1.08–1.64)	1.19 (0.96–1.48)	53.2 (48.8–57.5)	1.13 (0.93–1.37)	1.06 (0.86–1.30)
Midwest	59.1 (56.6–61.5)	1.10 (0.97–1.26)	1.13 (0.98–1.29)	55.7 (53.4–58.0)	1.25 (1.10–1.42)	1.25 (1.10–1.43)
Northeast	64.3 (61.4–67.1)	1.37 (1.18–1.60)	1.30 (1.11–1.52)	63.4 (60.6–66.2)	1.72 (1.48–2.00)	1.68 (1.44–1.96)
**Insurance status**						
Uninsured	44.8 (37.0–52.9)	Ref	Ref	39.6 (31.7–48.0)	Ref	Ref
Any Medicaid	63.2 (60.3–66.1)	2.12 (1.50–3.00)	2.24 (1.55–3.24)	58.3 (55.4–61.1)	2.14 (1.49–3.07)	2.02 (1.40–2.90)
Private insurance only	59.6 (57.6–61.4)	1.81 (1.30–2.53)	1.68 (1.16–2.42)	53.4 (51.5–55.2)	1.75 (1.23–2.48)	1.53 (1.05–2.22)
Other insurance	56.6 (51.4–61.8)	1.61 (1.09–2.37)	1.62 (1.09–2.42)	48.5 (43.0–54.1)	1.44 (0.95–2.17)	1.35 (0.87–2.11)
**Poverty status, % of poverty line**						
Below poverty	62.9 (58.9–66.7)	Ref	Ref	58.8 (54.7–62.8)	Ref	Ref
Above poverty ≤USD 75 k	56.4 (53.5–59.3)	0.76 (0.62–0.94)	0.84 (0.67–1.07)	51.8 (49.0–54.6)	0.75 (0.62–0.92)	0.73 (0.58–0.92)
Above poverty >USD 75 k	61.2 (59.2–63.1)	0.93 (0.77–1.12)	1.00 (0.75–1.33)	54.0 (52.0–55.9)	0.82 (0.68–0.99)	0.80 (0.62–1.04)
**Mother’s education status**						
College graduate	62.3 (60.3–64.2)	Ref	Ref	55.7 (53.7–57.6)	Ref	Ref
Some college	56.1 (53.2–59.0)	0.78 (0.67–0.90)	0.75 (0.64–0.88)	53.2 (50.2–56.2)	0.90 (0.78–1.04)	0.85 (0.72–1.00)
High school only	56.9 (53.0–60.8)	0.80 (0.67–0.96)	0.78 (0.63–0.97)	50.6 (46.7–54.5)	0.82 (0.68–0.97)	0.74 (0.60–0.90)
Less than high school	65.9 (60.2–71.2)	1.17 (0.90–1.52)	1.11 (0.80–1.55)	57.6 (52.2–62.9)	1.08 (0.86–1.37)	0.94 (0.70–1.27)
**Provider recommendation**						
No	34.2 (30.6–38.0)	Ref	Ref	31.9 (28.8–35.2)	Ref	Ref
Yes	64.8 (63.2–66.4)	1.88 (1.72–2.06)	1.92 (1.75–2.11)	60.8 (59.1–62.4)	1.82 (1.67–1.97)	1.81 (1.67–1.97)
**Survey year**						
2019	57.1 (54.8–59.5)	Ref	Ref	52.1 (49.9–54.4)	Ref	Ref
2020	62.9 (61.1–64.8)	1.27 (1.12–1.44)	1.28 (1.12–1.45)	56.2 (54.3–58.2)	1.18 (1.04–1.33)	1.17 (1.03–1.33)

w% = weighted percentages; OR = odds ratio; CI = confidence interval; Ref = reference category. UTD = up-to-date was defined as 3+ human papillomavirus shots (9 V, 4 V, UV, CV, or HP) or 2+ human papillomavirus shots, with first shot received before age 15 and interval between first and second shots of at least 5 months (minus 4 days), excluding any vaccinations after the random digit dialing interview date. ^a^ Model adjusted for sociodemographic characteristics, provider recommendation, and survey year.

## Data Availability

Data supporting reported results can be found at https://www.cdc.gov/vaccines/imz-managers/nis/datasets-teen.html, accessed on 5 April 2022.
